# Evolutionary Background of Precise Vocal–Respiratory Temporal Coordination in Human Infants

**DOI:** 10.1111/nyas.70409

**Published:** 2026-09-30

**Authors:** Miki Takahasi, Reiko Mazuka

**Affiliations:** ^1^ RIKEN Center for Brain Science Saitama Japan; ^2^ Research Institute for Science and Engineering Waseda University Tokyo Japan; ^3^ International Research Institute for Neurointelligence University of Tokyo Tokyo Japan; ^4^ Department of Psychology and Neuroscience Duke University Durham North Carolina USA

**Keywords:** human infants, laryngeal–respiratory coordination, swallowing, vocal development, vocal timing control

## Abstract

Human infants acquire speech through prolonged postnatal development. Sequential vocalizations, including speech, can be viewed as motor patterns consisting of alternating sound and silence. This temporal structure depends on the coordination of respiratory airflow and laryngeal activity, yet relatively little attention has been paid to how such timing control develops in infancy. In this review, we examine the development of vocal timing across two contexts: spontaneous vocalizations produced in nonsocial settings and vocal exchanges during face‐to‐face interactions. We highlight a developmental transition around the second half of the first year, in which vocal timing first becomes relatively stabilized in relation to respiratory maturation and is then increasingly used flexibly in social interaction. To place this transition in an evolutionary context, we review comparative findings from songbirds and marmosets and consider swallowing as another vital behavior requiring tight laryngeal–respiratory coordination. In humans, rapid postnatal brain growth may promote early complementary feeding, increasing the need for flexible regulation of swallowing timing, while laryngeal descent may heighten the need for airway protection. These pressures may have provided a physiological background for the later elaboration of flexible vocal timing control.

## Graphical abstract

In humans, early complementary feeding may have increased demands on flexible regulation of swallowing timing, while laryngeal descent may have increased the cost of failure by heightening the need for airway protection. These pressures may have promoted early laryngeal–respiratory coordination, providing a physiological context for the later elaboration of flexible vocal timing control.

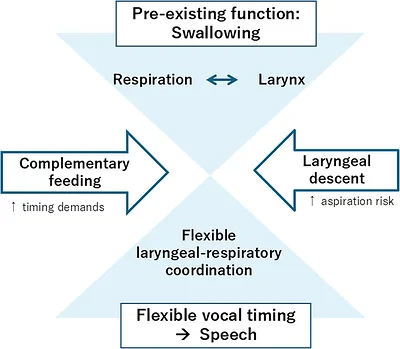

## 1 Introduction

Thousands of languages are spoken around the world, each with its own distinctive vocabulary and grammatical systems. Despite this diversity, a universal feature of humans is that infants begin to acquire speech as a means of communication soon after birth. In human infants, the first recognizable word typically emerges at approximately 1 year of age, whereas multiword utterances usually begin during the second year of life and continue to develop gradually thereafter [1]. Speech can also be viewed as a motor behavior, and its temporal regulation may develop in parallel with linguistic development. In this review, we focus on the development of vocal timing control in human infants.

At the most basic level, vocal production relies on vocal fold vibration driven by expiratory airflow. Although the resulting acoustic signals are shaped by the vocal tract and by articulatory movements of the tongue, lips, and other structures [2, 3], sequential vocalizations, including speech, consist of alternating periods of sound and silence. These alternating periods are made possible by the coordinated initiation and cessation of vocal fold vibration and expiratory airflow. Humans can control this temporal structure, organizing alternations between sound and silence through the precise coordination of multiple vocal‐related organs. In particular, coordination between the laryngeal and respiratory systems plays an important role in sequential vocalizations. From this perspective, vocal development may involve the gradual organization of laryngeal–respiratory coordination.

At the same time, the organs involved in vocal production serve vital functions. Respiration is essential for gas exchange, and laryngeal control is essential for airway protection during swallowing. Dysfunction of these systems can have serious consequences for health. These systems are primarily regulated by reflexive and autonomic mechanisms [4, 5]. In humans, however, they can also be voluntarily and precisely controlled during vocalization. This raises an evolutionary question: what selective pressures might have shaped the early development of precise and flexible laryngeal–respiratory coordination in human infancy? In this review, we examine the evolutionary background of this coordination by integrating findings from human infant development and comparative studies in songbirds and marmosets. We first focus on developmental changes in the temporal structure of infant vocalizations across two contexts: (i) spontaneous vocalizations and (ii) face‐to‐face vocal interactions between infants and their caregivers. We then consider analogous developmental processes in other vocal species, reviewing songbird vocal development as a model of self‐generated vocal practice and marmoset vocal turn‐taking as a model of socially coordinated vocal timing. In addition, we review current physiological and neural evidence relevant to vocal timing control in humans, songbirds, and marmosets. Finally, we discuss the evolutionary background of vocal timing control.

## 2 Development of the Temporal Structure of Vocalizations

### 2.1 Development of Temporal Structure in Spontaneous Vocalizations

Extensive research has examined how infant vocalizations change during the first year of life. Descriptive studies of early vocal development have provided an important framework for characterizing age‐related changes in vocal forms, as reviewed by Vihman [1]. Oller and colleagues highlighted the functional flexibility of protophones, a class of infant vocalizations that are not rigidly tied to specific emotional states or contexts [6, 7, 8]. Protophones take a variety of forms, including vocants, squeals, growls, and later canonical babbling. This concept is important for considering the evolutionary origins of speech, because functional flexibility is a key property that distinguishes speech from reflexive or affect‐bound vocalizations. This broader view motivates a focus not only on what kinds of vocalizations infants produce but also on how vocalizations are temporally organized into sequences of sound and silence in spontaneous vocal activity and mother–infant interactions. In this section, we review research that has explicitly investigated vocal–respiratory coordination, vocal–motor coordination, and the development of temporal organization across sequences of vocalizations.

The respiratory system itself undergoes substantial anatomical and physiological changes during infancy. For example, lung volume increases, as reviewed in [9], and quiet breathing patterns gradually change [10]. As the respiratory system matures, respiratory control for vocalizations is also likely to change [9, 11, 12]. Wilder and Baken [13] recorded crying and respiratory activity in infants aged 0–8 months. They reported that the duration of the inspiratory phase remained constant across age, whereas that of the expiratory phase, which provides the airflow used for vocal production, increased with age. Furthermore, respiratory kinematic movements during vocalization show patterns that differ from those observed during quiet breathing and also show developmental change [14, 15]. These developmental changes in respiratory control during vocalization are observed through the first year of life [16], whereas associations with specific vocal types are not clearly observed in the second year [16, 17]. These findings suggest that vocal–respiratory coordination develops during the prelinguistic period as the respiratory system matures physiologically.

Using longitudinal audio recordings collected from birth onward from five infants [18], Takahasi et al. [19] analyzed the developmental trajectory of the temporal structure of nonemotional spontaneous vocalizations. In their analysis, vocal duration and silent intervals were quantified as note duration and inter‐note interval, respectively (Figure 1), demonstrating that the average vocal duration gradually increased up to approximately 6 months of age, but remained relatively stable thereafter. In contrast, the silent intervals between successive vocalizations remained constant after birth. Furthermore, after 6 months of age, the average duration of vocalization and silent intervals converged to similar ranges. At the level of individual vocal units, the local proportions of vocal duration and the preceding and following silent intervals were nearly equivalent. These findings indicate that a stable balance between vocal duration and silent intervals emerges before the onset of first words, despite variation in vocal types and acoustic characteristics (Figure 1).

**FIGURE 1:**
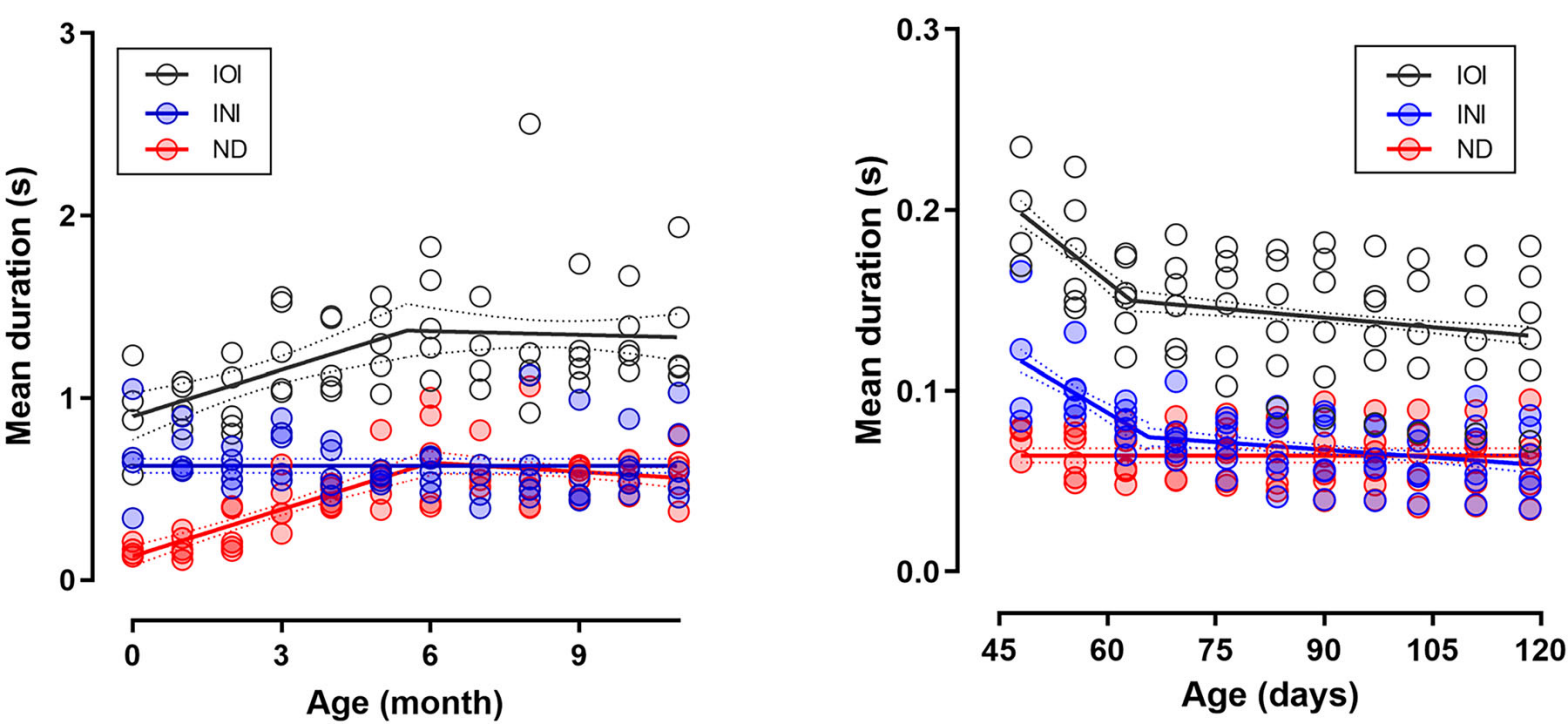
Developmental changes in temporal parameters of vocalizations. Developmental changes in the temporal parameters of vocalizations in human infants (left panel) and Bengalese finches (right panel). In the early phase, note duration (ND) was shorter than inter‐note interval (INI) in both species. As development progressed, ND and INI converged in both species. Nonetheless, the temporal parameters exhibiting developmental changes differ between species. In human infants, ND increases until approximately 6 months of age, whereas in Bengalese finches, INI shortens by approximately 70 days posthatching. Each circle represents the mean value for an individual, and the colors indicate different temporal parameters. The graphs are modified from those of [19].

As infants continue to develop, they modify the timing of their vocalizations in social contexts and begin to engage in vocal exchanges with others. The temporal structure established through spontaneous vocalizations thus may provide a foundation for conversational timing patterns later in life. In the following section, we describe how this early form of vocal timing develops into temporal coordination during face‐to‐face interactions.

### 2.2 Development of Temporal Structure in Face‐to‐Face Vocal Interactions

In adult conversations, the average time required for speaker change is approximately 250 ms, and this latency is shared across multiple languages [20]. Conversational timing continues to develop throughout childhood. For example, average response latencies in preschool children range from approximately 0.8 to 1.8 s, substantially longer than those observed in adults [21, 22]. By approximately 11 months of age, infants produce vocalizations while directing their gaze toward social partners, which have been interpreted as communicative vocalizations in interpersonal contexts, even though the vocalizations remain nonlinguistic [23]. Going further back in development, proto‐conversational turn‐taking with caregivers can be observed as early as approximately 3 months of age.

Gratier et al. [24] analyzed patterns of proto‐conversational exchanges between mothers and infants around 3 months of age. They found that infants responded to maternal vocalizations with a range of timing patterns, including vocal overlap, immediate responses following maternal offset (latching), and responses separated by longer gaps. Extending this work, Hilbrink et al. [25] examined developmental changes in response latencies between mothers and infants. They reported that infant response latencies to maternal vocalizations remained relatively constant at approximately 600 ms until 5 months of age, increased transiently up to 9 months, and then shortened again toward 18 months of age. Additionally, the frequency of vocal overlapping with maternal speech decreased with age. In contrast, maternal response latencies remained stable across infant age.

Previous accounts of early dialogue have emphasized the adult role in sustaining interactions by waiting for a child's response [26]. However, the findings reported by Gratier et al. and Hilbrink et al. suggest that infant response timing itself changes substantially across the first year of life, whereas maternal response timing remains relatively stable. These findings suggest that the temporal organization of vocal interactions reflects developmental changes in infant response timing rather than caregiver timing alone.

### 2.3 A Developmental Shift in Vocal Control Viewed Through Temporal Structure

The evidence reviewed above highlights a developmental transition in vocal timing around the second half of the first year. In nonsocial contexts, major changes in spontaneous vocalizations occur up to approximately 6 months of age, characterized mainly by the lengthening of vocal segments while silent intervals remain relatively stable. This pattern suggests that early changes in vocal timing may primarily reflect infants’ increasing ability to sustain vocalization within the expiratory phase. This interpretation is consistent with the broader literature on vocal and speech breathing in infants and adults, which shows that vocal production is supported by an asymmetric respiratory cycle with relatively short inspirations and prolonged expirations used for phonation [11, 13, 27]. In interactive contexts, marked changes in vocal timing appear after approximately 6 months of age, as infants increasingly adjust the timing of their vocal responses in relation to adult vocalizations. Viewed from the perspective of temporal structure, this transition may reflect a shift from the stabilization of self‐organized laryngeal–respiratory coordination to the flexible social use of this coordination (Figure 2).

**FIGURE 2:**
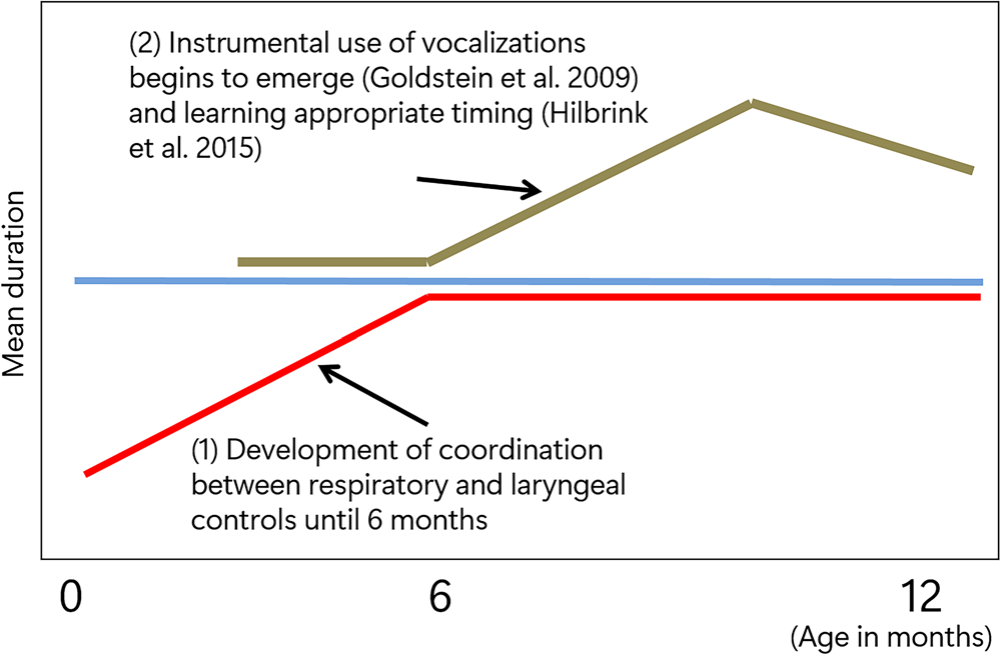
Developmental shift in temporal control of infant vocalizations across contexts. Schematic illustration of the proposed developmental shift in the control of temporal parameters of infant vocalizations across the two contexts. (1) Once coordination between laryngeal and respiratory control stabilizes during early infancy, (2) vocalizations begin to be used instrumentally as tools to elicit social responses and regulate interaction timing.

Support for this interpretation comes from studies of social contingency, including work using the still‐face paradigm, which was originally developed to examine infant social development [28]. Gros‐Louis et al. [29] reported that mothers respond to infants’ prelinguistic vocalizations at a rate of approximately 30%–50%. Focusing on situations in which caregivers respond contingently to infant vocalizations, Goldstein and colleagues demonstrated that when mothers temporarily stopped providing facial expressions and vocal feedback during ongoing interactions (still‐face episodes), infants showed a transient increase in vocal production rate [30]. Notably, this increase was observed in 5‐month‐old infants but not in 2‐month‐old infants [31].

Adults’ contingent responses have been shown to increase both the quantity of infant vocalizations and the quality of their acoustic and structural properties, such as the production of consonant–vowel combinations [32, 33, 34]. By contrast, relatively few studies have directly examined how contingent responses influence the temporal control of vocalizations, such as response timing or latency. The developmental changes in response latency reported by Hilbrink et al. suggest that infants may explore different ways of regulating the timing of their vocalizations through interactions with caregivers. Direct experimental evidence for this process, however, remains an important topic for future research.

## 3 How Is Vocal Control Achieved? Comparative Studies in Songbirds and Marmosets

To understand how precise vocal timing control evolved, comparative evidence from other vocal‐learning species is informative. Songbirds are a well‐established model for vocal learning, and extensive research has revealed how vocal production is coordinated with respiration during song development. We also review recent findings from marmosets, a primate species that exhibits rich vocal interactions and socially influenced vocal development. Together, these studies provide insights into the developmental and evolutionary foundations of vocal control.

### 3.1 Vocal–Respiratory Coordination in Songbirds

Vocal production is generated through the coordinated activity of expiratory airflow as a power source, a vocal organ as a sound source, and articulatory organs such as the tongue, jaw, and pharyngeal structures as a filter (the source–filter model) [2, 3]. Vocal learning refers to the ability to acquire novel vocal repertoires through experience [35]. To produce learned vocalizations, animals must acquire motor patterns that precisely coordinate respiratory, phonatory, and articulatory systems.

Songbirds possess specialized neural circuits for song production and song learning [36, 37, 38]. The song motor pathway includes the forebrain nuclei HVC and RA (robust nucleus of the arcopallium), which are essential for song production. HVC projects to RA, and RA in turn projects to brainstem vocal and respiratory nuclei, including the nucleus retroambigualis (RAm) and the tracheosyringeal portion of the hypoglossal nucleus (nXIIts), thereby directly linking forebrain activity to respiratory and vocal motor output. In parallel, the anterior forebrain pathway, a cortico–basal ganglia circuit involved in song learning, forms an indirect loop connecting HVC and RA through Area X, the dorsolateral nucleus of the medial thalamus (DLM), and the lateral magnocellular nucleus of the anterior nidopallium (LMAN).

Developmental studies further revealed that vocal production can emerge before mature vocal–respiratory coordination is established. At the beginning of song development, juvenile songbirds produce highly variable immature vocalizations known as subsongs [39, 40]. Experimental lesion studies showed that subsong production depends primarily on LMAN, whereas HVC in the song motor pathway is not yet required at this stage [41]. Vocal–respiratory coordination during subsong production is also immature, and the onset and offset of vocalizations are not consistently synchronized with expiratory airflow. As song development progresses, vocal–respiratory coordination gradually becomes more precise. Lesion studies further demonstrated that damage to HVC disrupts the synchronization between respiration and vocal output, suggesting that HVC plays a critical role in coordinating the temporal relationship between breathing and vocal production [42].

In songbirds, direct projections exist from the forebrain song motor nucleus RA to both RAm and nXIIts in the brainstem. In contrast, closely related suboscine species that are not vocal learners show direct projections from the RA homolog area to RAm but lack direct projections to nXIIts [43]. These findings suggest that parallel cortical control of both respiratory and sound‐source motor nuclei is critical for vocal learning [43].

Songbird studies highlight two important principles regarding the evolution of voluntary vocal control. First, vocal–respiratory coordination is not present from the onset of vocal production but develops through sensorimotor learning. Once established, this coordination depends on forebrain motor circuits, particularly HVC. Second, vocal learning is associated with parallel forebrain projections to both respiratory and sound‐producing motor nuclei, suggesting that coordinated descending control of these systems is important for learned vocal behavior.

### 3.2 Vocal–Respiratory Coordination Through Turn‐Taking in Marmosets

Marmosets are New World monkeys that diverged early from the human lineage. However, they share some social characteristics with humans, including cooperative breeding, in which not only mothers but also other group members participate in infant care [44]. Marmosets produce phee calls to maintain contact with other individuals [45]. During development, phee calls gradually emerge from immature vocalizations called cries. This developmental transition is influenced by both vocal interactions with adults and physiological development of the larynx.

Analyses of vocal exchanges between infants and adults showed that infants whose calls received more contingent responses from adults transitioned more rapidly from cries to phee calls [46, 47]. At the same time, the marmoset larynx changes in mass and tissue elasticity. These morphological changes facilitate the production of phee calls and contribute to nonlinear developmental changes in vocal behavior [48]. Also, laryngeal and expiratory muscle activities are synchronized during phee calls, and a coupled oscillator model of laryngeal–respiratory control can account for the developmental emergence of this coordination [48]. This developmental pattern parallels findings from songbirds and human infants, in which mature vocal production emerges together with increasingly precise vocal–respiratory coordination [13, 42]. In addition, phee call production is coordinated not only with respiration and laryngeal activity but also with autonomic physiological processes, including cardiac and blood flow rhythms [49].

Taken together, marmoset studies suggest that primate vocal development involves not only maturation of the larynx and respiration but also the coordination of vocal production with autonomic physiological processes and social feedback. These findings broaden the question raised by songbird studies. In songbirds, learned vocal production is supported by specialized forebrain projections to respiratory and vocal motor nuclei. In primates, however, the neural organization underlying flexible vocal timing is less clearly understood. Direct corticobulbar projections from the laryngeal motor cortex to the nucleus ambiguus may contribute to voluntary laryngeal control in humans [50, 51], but such projections are not entirely unique to humans and appear to be sparse in nonhuman primates [52]. Moreover, marmoset vocalizations are embedded in broader physiological dynamics, including respiratory, laryngeal, cardiac, and blood flow rhythms [49]. Thus, primate vocal control is unlikely to depend on direct cortical control of the larynx alone. Rather, it may require the developmental refinement of broader brainstem and autonomic networks that coordinate respiration, laryngeal activity, and physiological state.

### 3.3 Other Functions Requiring Coordination Between Laryngeal Activity and Respiration

Building on the songbird and marmoset studies reviewed above, a further question arises: what developmental or evolutionary pressures could have promoted early coordination among respiration, laryngeal control, and brainstem physiological regulation in humans? Vocal control may be viewed as one aspect of the broader refinement of interactions between voluntary motor control and autonomic physiological regulation. However, vocal control differs from many other developing voluntary motor behaviors in one important respect: it directly involves organs that are essential for survival. Flexible control of these systems may therefore entail risks, because poorly coordinated vocalization could interfere with breathing and airway protection.

To address this issue, we turn to another vital function that uses partly overlapping organs and neural circuits: swallowing. Like vocalizations, swallowing requires tight coordination between laryngeal activity and respiration. Unlike vocalization, swallowing must function from early infancy to maintain nutrition and protect the airway (Figure 3).

**FIGURE 3:**
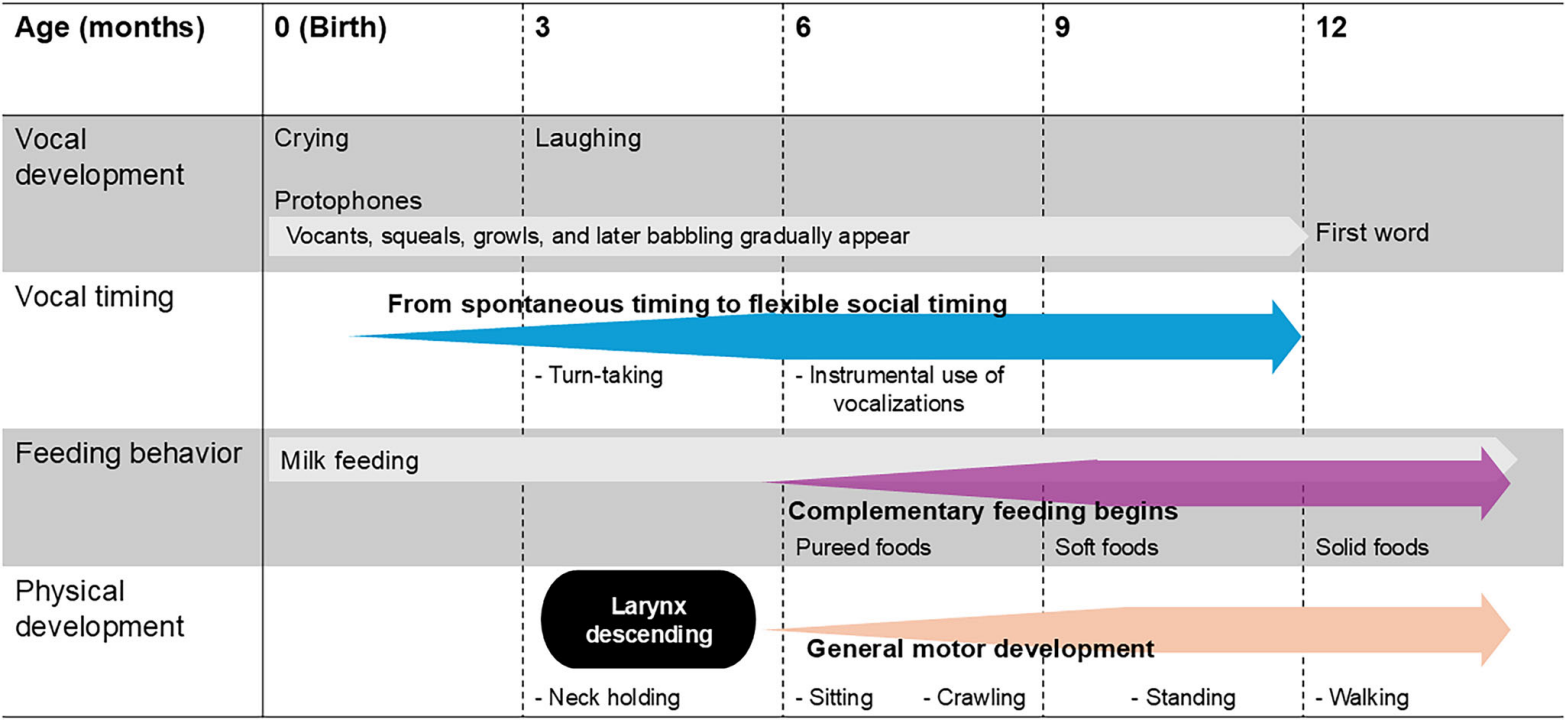
Developmental timeline of vocal timing, swallowing, feeding, and general motor development in human infancy. Early infant vocalizations, including protophones, are present from the beginning of life. The figure illustrates parallel developmental changes in vocal timing, feeding, laryngeal descent, and general motor development. Around the second half of the first year, complementary feeding introduces new oral sensorimotor demands, while infants increasingly use vocal timing flexibly in social interaction.

In many mammals, the respiratory and digestive pathways share the pharyngeal region, creating a risk that food or liquid may enter the airway. Airway protection during feeding is therefore supported by multiple safeguards, including anatomical structures such as the epiglottis and dynamic mechanisms such as laryngeal closure, temporary respiratory inhibition, and expiratory resumption after the swallow [53, 54]. In human infants, these protective mechanisms operate within a rapidly changing developmental context [55]. During the first months of life, changes in feeding behavior, respiratory control, and laryngeal position, including the beginning of laryngeal descent [56], increase the need for precise coordination between swallowing and respiration. Consistent with this view, the timing of swallowing becomes increasingly organized in relation to the respiratory cycle during early infancy [57, 58].

The motor pattern for swallowing is generated primarily by brainstem networks involving the nucleus tractus solitarius and nucleus ambiguus, which interact closely with respiratory control circuits [59]. However, swallowing is not purely reflexive. In human adults, cortical regions involved in swallowing and vocalization are located in nearby ventral sensorimotor areas [59]. Electrical stimulation of the primary motor cortex in nonhuman primates can evoke swallowing movements, indicating that descending cortical influences can act on swallowing‐related brainstem circuits in primates [60]. Thus, at least in primates, cortical processes can influence brainstem networks coordinating laryngeal and respiratory activity during swallowing.

Swallowing requires laryngeal–respiratory coordination from early infancy, and this coordination is based primarily on brainstem networks while also being subject to cortical modulation. Because vocal production also depends on coordinated laryngeal and respiratory activity, swallowing provides a useful comparison for considering how shared peripheral systems can be organized for different behavioral goals. Possible links among ingestion‐related movements, facial expressions, and vocal communication have long been discussed in primate studies [61]. More recent work in macaques showed that perioral muscle activity differs between ingestive behaviors, such as chewing, sucking, and drinking, and communicative behaviors, such as lip‐smacking [62]. These findings suggest that overlapping peripheral structures can be organized into distinct behavior‐specific motor patterns depending on behavioral context. The hierarchical framework proposed by Zhang and Ghazanfar [63] further suggests that such context‐dependent organization may depend on interactions among respiratory, laryngeal, orofacial, and autonomic processes under higher‐order control. This framework may also be useful for considering the relationship between swallowing and vocalization: both behaviors rely partly on overlapping laryngeal and respiratory systems, but they are organized for different behavioral goals and may receive different forms of modulation. In humans, swallowing may be especially important in this comparison because airway protection places survival‐related demands on laryngeal–respiratory coordination from early infancy.

## 4 Why Does Laryngeal–Respiratory Coordination During Swallowing Develop So Early in Humans?

Swallowing is essential for nutrition; however, it also entails a life‐threatening risk of aspiration. Previous accounts of speech evolution have emphasized the importance of enhanced respiratory control for vocal production [64]. Here, we address a complementary question: why precise coordination between the respiratory and laryngeal systems became important so early in human development. We propose that two interacting evolutionary pressures associated with swallowing contributed to the early importance of laryngeal–respiratory coordination. First, rapid postnatal brain growth and early complementary feeding required infants to adjust swallowing timing under conditions of still‐developing oral motor control, as the transition from sucking‐based feeding to chewing and solid‐food processing changed the sensory and motor demands within the oral cavity. Second, permanent laryngeal descent increased the cost of failure in this coordination by heightening the risk of aspiration. Thus, early complementary feeding increased the need for flexible sensorimotor regulation of swallowing timing, whereas laryngeal descent increased the need for reliable airway protection.

### 4.1 Prolonged Infancy and Early Weaning

Across mammals, lactation and weaning are closely linked to offspring growth, food quality, and the maturation of digestive function [65]. Compared with other primates, humans have an unusually prolonged period of dependency before children become fully independent in feeding and other aspects of daily life. Paradoxically, however, the introduction of complementary foods begins relatively early. In many nonhuman primates, the transition toward weaning typically occurs after infants have attained sufficient body growth and after molar eruption has begun. In humans, by contrast, complementary feeding commonly begins at around 6 months of age, when infants still lack molars and often do not yet have a complete set of incisors [66].

Human infants undergo pronounced postnatal growth, especially of the brain. Recent comparative analyses suggest that apparent human altriciality is driven less by exceptional neonatal brain immaturity than by marked enlargement of the brain after birth [67]. The metabolic costs of postnatal brain growth and maintenance may constrain the duration of exclusive breastfeeding, because the energetic requirements of large‐brained infants can exceed what mothers can provide through milk alone at around 6 months [66]. The introduction of complementary foods around 6 months may therefore have provided an adaptive solution, allowing infants to obtain additional nutrition.

This transition changes the demands placed on swallowing. Sucking‐based feeding relies on relatively stereotyped coordination among sucking, swallowing, and respiration. By contrast, the introduction of complementary foods requires infants to process materials with more variable textures and to begin coordinating oral movements related to chewing and bolus formation. In adults, volitional swallowing involves cortical sensorimotor networks, indicating that swallowing can be modulated by higher‐order sensorimotor processes [68, 69]. Under these conditions, swallowing cannot rely solely on a fixed sucking–swallowing pattern; its timing must be adjusted according to the sensory and motor state of the oral cavity. Consistent with the idea that feeding transitions involve broader sensorimotor reorganization, studies in rodents have shown that the transition from sucking to chewing is accompanied by reorganization of oral sensorimotor cortical representations [70]. Although direct evidence from human infants remains limited, these findings support the idea that the developmental shift from sucking to chewing involves broader sensorimotor reorganization. Thus, early weaning may have increased the need for flexible regulation of swallowing timing, thereby strengthening developmental and evolutionary pressures on the coordination between laryngeal closure and respiration during early development.

### 4.2 Increased Aspiration Risk due to Laryngeal Descent

In humans, the larynx is positioned lower in the throat than in many other mammals [71, 72, 73]. In discussions of speech evolution, laryngeal descent has often been considered important because it reshapes the vocal tract and allows a wider range of formant patterns, thereby expanding the acoustic space available for speech sounds [74, 75]. Here, however, we focus on a different consequence of the same anatomical configuration: its cost for airway protection. A low laryngeal position increases the overlap between the respiratory and digestive pathways in the pharynx, thereby increasing the risk that food or liquid may enter the airway. In this configuration, swallowing and breathing must be precisely coordinated to maintain airway protection.

This anatomical condition differs from that in many other mammals, in which the larynx is positioned closer to the soft palate, helping to keep the respiratory and digestive pathways more clearly separated and reducing the risk of aspiration [56, 71]. Laryngeal descent has also been reported during development in chimpanzees, but the larynx later moves upward relative to the oral cavity as the face grows, unlike the permanent low position observed in humans [73, 76]. Thus, what is distinctive in humans is not simply that the larynx descends, but that a relatively low laryngeal position is maintained, imposing persistent demands on the coordination of swallowing, airway protection, and respiration.

Importantly, these demands arise early in infancy. During the first half of the first year, many voluntary motor skills are still undergoing rapid development. Nevertheless, swallowing must already be coordinated with laryngeal closure and respiration to maintain nutrition while protecting the airway. Thus, compared with many other developing voluntary motor behaviors, laryngeal–respiratory coordination during swallowing must reach a functional level particularly early in life.

## 5 Conclusion

In this review, we examined the development of vocal timing control in human infants and considered why coordination between the respiratory and laryngeal systems, which are essential for survival, becomes available so early in development. Developmental studies of infant vocalizations suggest a shift around the second half of the first year: vocal timing first becomes relatively stabilized in relation to respiratory maturation and is then increasingly used flexibly in social interaction. Comparative studies in songbirds and marmosets further suggest that vocal timing control is shaped by both neural maturation and social experience. These findings support the view that human infant vocal development should be understood not only as the emergence of language‐specific vocal forms but also as the development of motor control over sound and silence.

Previous accounts of speech evolution have emphasized enhanced respiratory control as a key component of human vocal capacity [64]. Our proposal complements this view by asking why coordination between respiration and laryngeal control became important early in human development. We suggest that swallowing provides a useful evolutionary context for this question. In humans, the energetic demands of rapid postnatal brain growth may constrain the duration of exclusive breastfeeding and promote early complementary feeding, thereby increasing the need for flexible regulation of swallowing timing as infants shift from sucking‐based feeding to chewing and solid‐food processing. Laryngeal descent may further increase the cost of failure in this regulation by heightening the need for airway protection. Together, these pressures may have promoted the early establishment of functional laryngeal–respiratory coordination for swallowing, providing an important physiological background for the later elaboration of flexible vocal timing control.

This perspective also provides a possible link between human development and comparative findings from marmosets. Marmosets and humans differ greatly in phylogeny and vocal‐learning capacity, yet both species exhibit cooperative breeding and socially influenced vocal development. More generally, species with costly parental care, especially those living in social groups, are expected to evolve mechanisms for parent–offspring recognition that reduce confusion over reproductive investment, including vocal recognition in some species [77]. In humans, early complementary feeding may have allowed nutritional care to be shared among caregivers, placing feeding within a broader social context of cooperative provisioning and extended dependency [78]. In marmosets, cooperative breeding may have evolved under different pressures, including the high energetic and caregiving demands associated with twin births. The need to maintain social contact within such caregiving systems may have favored early development of contact calls such as phee calls. Thus, although the evolutionary routes may differ, humans and marmosets suggest that vocal development is embedded in a broader context of physiological maturation, survival‐related behavior, and social caregiving.

We therefore propose that the origins of human vocal timing control should be considered within an integrated framework linking vocal development, swallowing, respiration, and social interaction. Human speech did not emerge from vocal learning alone, nor from respiratory control alone. Rather, it may have built on early‐developing coordination among vital systems that were already required for survival and later became available for flexible, socially organized vocal behavior. Future work should examine how feeding, respiration, and vocal interaction are coordinated during infancy, and whether caregiver responses in both feeding and vocal contexts contribute to the development of flexible laryngeal–respiratory control. Because the evidence for a developmental shift in spontaneous vocal timing is still based on a small longitudinal sample, larger datasets will be needed to determine how robust this pattern is across individual infants, languages, and social environments.

## Author Contributions

M.T. and R.M. were responsible for the conceptualization, literature review, and manuscript writing.

## Conflicts of Interest

The authors declare no conflicts of interest.

## References

[1] M. M. Vihman , Phonological Development, 2nd ed. (Wiley‐Blackwell, 2014), 10.1002/9781394260997.

[2] G. Fant , Acoustic Theory of Speech Production: With Calculations Based on X‐Ray Studies of Russian Articulations (Mouton de Gruyter, 1971), 10.1515/9783110873429.

[3] T. Riede and F. Goller , “Peripheral Mechanisms for Vocal Production in Birds—Differences and Similarities to Human Speech and Singing,” Brain and Language 115 (2010): 69–80, 10.1016/j.bandl.2009.11.003. PMC2896990 20153887

[4] B. M. Koeppen and B. A. Stanton , Berne & Levy Physiology, 8th ed., ed. J. M. Hall and A. Swiatecka‐Urban (Elsevier—Health Sciences Division, 2023).

[5] C. L. Ludlow , “Central Nervous System Control of Voice and Swallowing,” Journal of Clinical Neurophysiology 32 (2015): 294–303, 10.1097/WNP.0000000000000186. PMC4526113 26241238

[6] D. K. Oller , “The Emergence of the Sounds of Speech in Infancy,” in Child Phonology, ed. G. H. Yeni‐Komshian , J. F. Kavanagh and C. A. Ferguson (Elsevier, 1980), 93–112, 10.1016/b978-0-12-770601-6.50011-5.

[7] D. K. Oller , M. Caskey , H. Yoo , et al., “Preterm and Full Term Infant Vocalization and the Origin of Language,” Scientific Reports 9 (2019): 14734, 10.1038/s41598-019-51352-0. PMC6791864 31611607

[8] D. K. Oller , E. H. Buder , H. L. Ramsdell , A. S. Warlaumont , L. Chorna , and R. Bakeman , “Functional Flexibility of Infant Vocalization and the Emergence of Language,” Proceedings of the National Academy of Sciences 110 (2013): 6318–6323, 10.1073/pnas.1300337110. PMC3631625 23550164

[9] M. Di Cicco , A. Kantar , B. Masini , et al., “Structural and Functional Development in Airways Throughout Childhood: Children Are Not Small Adults,” Pediatric Pulmonology 56 (2021): 240–251, 10.1002/ppul.25169. 33179415

[10] A. A. Colin , M. E. Wohl , J. Mead , F. A. Ratjen , G. Glass , and A. R. Stark , “Transition From Dynamically Maintained to Relaxed End‐Expiratory Volume in Human Infants,” Journal of Applied Physiology 67 (1989): 2107–2111, 10.1152/jappl.1989.67.5.2107. 2600036

[11] S. Fuchs , E. Rubertus , L. L. Koenig , and A. Noiray , “Toward a Fuller Integration of Respiratory Rhythms Into Research on Infant Vocal and Motor Development,” Annals of the New York Academy of Sciences 1556 (2026): e70197, 10.1111/nyas.70197. PMC12880585 41649880

[12] R. D. Kent , “The Maturational Gradient of Infant Vocalizations: Developmental Stages and Functional Modules,” Infant Behavior and Development 66 (2022): 101682, 10.1016/j.infbeh.2021.101682. 34920296

[13] C. N. Wilder and R. J. Baken , “Respiratory Patterns in Infant Cry,” Human Communication 1 (1974): 18–34.

[14] K. P. Connaghan , C. A. Moore , and M. Higashakawa , “Respiratory Kinematics During Vocalization and Nonspeech Respiration in Children From 9 to 48 Months,” Journal of Speech, Language, and Hearing Research 47 (2004): 70–84, 10.1044/1092-4388(2004/007. PMC3984457 15072529

[15] K. J. Reilly and C. A. Moore , “Respiratory Movement Patterns During Vocalizations at 7 and 11 Months of Age,” Journal of Speech, Language, and Hearing Research 52 (2009): 223–239, 10.1044/1092-4388(2008/06-0215). PMC3974901 18695025

[16] C. A. Boliek , T. J. Hixon , P. J. Watson , and W. J. Morgan , “Vocalization and Breathing During the First Year of Life,” Journal of Voice 10 (1996): 1–22, 10.1016/S0892-1997(96)80015-4. 8653174

[17] D. F. Parham , E. H. Buder , D. K. Oller , and C. A. Boliek , “Syllable‐Related Breathing in Infants in the Second Year of Life,” Journal of Speech, Language, and Hearing Research 54 (2011): 1039–1050, 10.1044/1092-4388(2010/09-0106). PMC3078530 21173390

[18] S. Amano , K. Kato , and T. Kondo , “Development of Japanese Infant Speech Database and Speaking Rate Analysis,” paper presented at the 7th International Conference on Spoken Language Processing, ICSLP2002 ‐ INTERSPEECH 2002, Denver, CO, September 317–320, 2002.

[19] M. Takahasi , K. Okanoya , and R. Mazuka , “How Vocal Temporal Parameters Develop: A Comparative Study Between Humans and Songbirds, Two Distantly Related Vocal Learners,” Journal of Language Evolution 6 (2021): 26–36, 10.1093/jole/lzaa008.

[20] T. Stivers , N. J. Enfield , P. Brown , et al., “Universals and Cultural Variation in Turn‐Taking in Conversation,” Proceedings of the National Academy of Sciences 106 (2009): 10587–10592, 10.1073/pnas.0903616106. PMC2705608 19553212

[21] M. Casillas , S. C. Bobb , and E. Clark , “Turn‐Taking, Timing, and Planning in Early Language Acquisition,” Journal of Child Language 43 (2015): 1310–1337, 10.1017/S0305000915000689. 26603859

[22] C. Garvey and G. Berninger , “Timing and Turn Taking in Children's Conversations,” Discourse Processes 4 (1981): 27–57, 10.1080/01638538109544505.

[23] E. Donnellan , C. Bannard , M. L. McGillion , K. E. Slocombe , and D. Matthews , “Infants' Intentionally Communicative Vocalizations Elicit Responses From Caregivers and Are the Best Predictors of the Transition to Language: A Longitudinal Investigation of Infants' Vocalizations, Gestures and Word Production,” Developmental Science 23 (2020): e12843, 10.1111/desc.12843. 31045301

[24] M. Gratier , E. Devouche , B. Guellai , R. Infanti , E. Yilmaz , and E. Parlato‐Oliveira , “Early Development of Turn‐Taking in Vocal Interaction Between Mothers and Infants,” Frontiers in Psychology 6 (2015): 1167–1167, 10.3389/fpsyg.2015.01167. PMC4560030 26388790

[25] E. E. Hilbrink , M. Gattis , and S. C. Levinson , “Early Developmental Changes in the Timing of Turn‐Taking: A Longitudinal Study of Mother–Infant Interaction,” Frontiers in Psychology 6 (2015): 1492, 10.3389/fpsyg.2015.01492. PMC4586330 26483741

[26] J. Dunn and M. Shatz , “Becoming a Conversationalist Despite (or Because of) Having an Older Sibling,” Child Development 60 (1989): 399–410, 10.2307/1130985.

[27] D. H. McFarland , “Respiratory Markers of Conversational Interaction,” Journal of Speech, Language, and Hearing Research 44 (2001): 128–143, 10.1044/1092-4388(2001/012. 11218097

[28] E. Tronick , H. Als , L. Adamson , S. Wise , and T. B. Brazelton , “The Infant's Response to Entrapment Between Contradictory Messages in Face‐to‐Face Interaction,” Pediatrics 62 (1978): 403–403, 10.1542/peds.62.3.403. 632477

[29] J. Gros‐Louis , M. J. West , M. H. Goldstein , and A. P. King , “Mothers Provide Differential Feedback to Infants' Prelinguistic Sounds,” International Journal of Behavioral Development 30 (2006): 509–516, 10.1177/0165025406071914.

[30] M. H. Goldstein , J. A. Schwade , and M. H. Bornstein , “The Value of Vocalizing: Five‐Month‐Old Infants Associate Their Own Noncry Vocalizations With Responses From Caregivers,” Child Development 80 (2009): 636–644, 10.1111/j.1467-8624.2009.01287.x. PMC4151607 19489893

[31] S. L. Elmlinger , J. A. Schwade , L. Vollmer , and M. H. Goldstein , “Learning How to Learn From Social Feedback: The Origins of Early Vocal Development,” Developmental Science 26 (2023): e13296, 10.1111/desc.13296. 35737680

[32] K. Bloom , A. Russell , and K. Wassenberg , “Turn Taking Affects the Quality of Infant Vocalizations,” Journal of Child Language 14 (1987): 211–227, 10.1017/S0305000900012897. 3611239

[33] M. H. Goldstein , A. P. King , and M. J. West , “Social Interaction Shapes Babbling: Testing Parallels Between Birdsong and Speech,” Proceedings of the National Academy of Sciences 100 (2003): 8030–8035, 10.1073/pnas.1332441100. PMC164707 12808137

[34] M. H. Goldstein and J. A. Schwade , “Social Feedback to Infants' Babbling Facilitates Rapid Phonological Learning,” Psychological Science 19 (2008): 515–523, 10.1111/j.1467-9280.2008.02117.x. 18466414

[35] A. J. Doupe and P. K. Kuhl , “Birdsong and Human Speech: Common Themes and Mechanisms,” Annual Review of Neuroscience 22 (1999): 567–631, 10.1146/annurev.neuro.22.1.567. 10202549

[36] M. S. Brainard and A. J. Doupe , “Auditory Feedback in Learning and Maintenance of Vocal Behaviour,” Nature Reviews Neuroscience 1 (2000): 31–40, 10.1038/35036205. 11252766

[37] F. Nottebohm , T. M. Stokes , and C. M. Leonard , “Central Control of Song in the Canary, *Serinus canarius* ,” Journal of Comparative Neurology 165 (1976): 457–486, 10.1002/cne.901650405. 1262540

[38] S. W. Bottjer , E. A. Miesner , and A. P. Arnold , “Forebrain Lesions Disrupt Development but Not Maintenance of Song in Passerine Birds,” Science 224 (1984): 901–903, 10.1126/science.6719123. 6719123

[39] C. K. Catchpole and P. J. B. Slater , Bird Song: Biological Themes and Variations, 2nd ed. (Cambridge University Press, 2008), 10.1017/CBO9780511754791.

[40] P. Marler , “A Comparative Approach to Vocal Learning: Song Development in White‐Crowned Sparrows,” Journal of Comparative and Physiological Psychology 71 (1970): 1–25, 10.1037/h0029144.

[41] D. Aronov , A. S. Andalman , and M. S. Fee , “A Specialized Forebrain Circuit for Vocal Babbling in the Juvenile Songbird,” Science 320 (2008): 630–634, 10.1126/science.1155140. 18451295

[42] L. Veit , D. Aronov , and M. S. Fee , “Learning to Breathe and Sing: Development of Respiratory‐Vocal Coordination in Young Songbirds,” Journal of Neurophysiology 106 (2011): 1747–1765, 10.1152/jn.00247.2011. PMC3191841 21697438

[43] W.‐C. Liu , K. Wada , E. D. Jarvis , and F. Nottebohm , “Rudimentary Substrates for Vocal Learning in a Suboscine,” Nature Communications 4 (2013): 2082–2082, 10.1038/ncomms3082. 23823977

[44] L. J. Digby , S. F. Ferrari , and W. Saltzman , “Callitrichines: The Role of Competition in a Cooperatively Breeding Species,” in Primates in Perspective, ed. C. J. Campbell , A. Fuentes , K. C. MacKinnon , M. A. Panger , and S. K. Bearder (Oxford University Press, 2007), 85–105.

[45] B. M. Bezerra and A. Souto , “Structure and Usage of the Vocal Repertoire of *Callithrix jacchus* ,” International Journal of Primatology 29 (2008): 671–701, 10.1007/s10764-008-9250-0.

[46] D. Y. Takahashi , A. R. Fenley , Y. Teramoto , et al., “The Developmental Dynamics of Marmoset Monkey Vocal Production,” Science 349 (2015): 734–738, 10.1126/science.aab1058. 26273055

[47] D. Y. Takahashi , D. A. Liao , and A. A. Ghazanfar , “Vocal Learning via Social Reinforcement by Infant Marmoset Monkeys,” Current Biology 27 (2017): 1844.e6–1852.e6, 10.1016/j.cub.2017.05.004. 28552359

[48] Y. S. Zhang , D. Y. Takahashi , D. A. Liao , A. A. Ghazanfar , and C. P. H. Elemans , “Vocal State Change Through Laryngeal Development,” Nature Communications 10 (2019): 4592, 10.1038/s41467-019-12588-6. PMC6785551 31597928

[49] J. I. Borjon , D. Y. Takahashi , D. C. Cervantes , and A. A. Ghazanfar , “Arousal Dynamics Drive Vocal Production in Marmoset Monkeys,” Journal of Neurophysiology 116 (2016): 753–764, 10.1152/jn.00136.2016. PMC6208312 27250909

[50] U. Jürgens , “Neural Pathways Underlying Vocal Control,” Neuroscience and Biobehavioral Reviews 26 (2002): 235–258, 10.1016/s0149-7634(01)00068-9. 11856561

[51] K. Simonyan , “The Laryngeal Motor Cortex: Its Organization and Connectivity,” Current Opinion in Neurobiology 28 (2014): 15–21, 10.1016/j.conb.2014.05.006. PMC4177508 24929930

[52] A. R. Lameira , “Bidding Evidence for Primate Vocal Learning and the Cultural Substrates for Speech Evolution,” Neuroscience & Biobehavioral Reviews 83 (2017): 429–439, 10.1016/j.neubiorev.2017.09.021. 28947156

[53] K. Matsuo and J. B. Palmer , “Coordination of Mastication, Swallowing and Breathing,” Japanese Dental Science Review 45 (2009): 31–40, 10.1016/j.jdsr.2009.03.004. PMC2749282 20161022

[54] C. T. Sasaki and E. M. Weaver , “Physiology of the Larynx,” The American Journal of Medicine 103 (1997): 9S–18S, 10.1016/s0002-9343(97)00314-8. 9422616

[55] N. Keven and K. A. Akins , “Beyond Neonatal Imitation: Aerodigestive Stereotypies, Speech Development, and Social Interaction in the Extended Perinatal Period,” Behavioral and Brain Sciences 40 (2017): e403, 10.1017/S0140525x17001923. 29342817

[56] C. T. Sasaki , P. A. Levine , J. T. Laitman , and E. S. Crelin , “Postnatal Descent of the Epiglottis in Man: A Preliminary Report,” Archives of Otolaryngology—Head and Neck Surgery 103 (1977): 169–171, 10.1001/archotol.1977.00780200095011. 836246

[57] B. N. Kelly , M.‐L. Huckabee , R. D. Jones , and C. M. A. Frampton , “The First Year of Human Life: Coordinating Respiration and Nutritive Swallowing,” Dysphagia 22 (2007): 37–43, 10.1007/s00455-006-9038-3. 17221292

[58] I. H. Gewolb and F. L. Vice , “Maturational Changes in the Rhythms, Patterning, and Coordination of Respiration and Swallow During Feeding in Preterm and Term Infants,” Developmental Medicine & Child Neurology 48 (2006): 589–594, 10.1111/j.1469-8749.2006.tb01320.x. 16780629

[59] N. Y. K. Li‐Jessen and C. Ridgway , “Neuroanatomy of Voice and Swallowing,” in Neurologic and Neurodegenerative Diseases of the Larynx, ed. P. Weissbrod and D. Francis (Springer, 2020): 21–40, 10.1007/978-3-030-28852-5_3.

[60] R. E. Martin , P. Kemppainen , Y. Masuda , D. Yao , G. M. Murray , and B. J. Sessle , “Features of Cortically Evoked Swallowing in the Awake Primate (*Macaca fascicularis*),” Journal of Neurophysiology 82 (1999): 1529–1541, 10.1152/jn.1999.82.3.1529. 10482767

[61] R. J. Andrew , “The Origin and Evolution of the Calls and Facial Expressions of the Primates,” Behaviour 20 (1963): 1–107, 10.1163/156853963x00220.

[62] S. V. Shepherd , M. Lanzilotto , and A. A. Ghazanfar , “Facial Muscle Coordination in Monkeys During Rhythmic Facial Expressions and Ingestive Movements,” The Journal of Neuroscience 32 (2012): 6105–6116, 10.1523/JNEUROSCI.6136-11.2012. PMC3353726 22553017

[63] Y. S. Zhang and A. A. Ghazanfar , “A Hierarchy of Autonomous Systems for Vocal Production,” Trends in Neurosciences 43 (2020): 115–126, 10.1016/j.tins.2019.12.006. PMC7213988 31955902

[64] A. M. MacLarnon and G. P. Hewitt , “The Evolution of Human Speech: The Role of Enhanced Breathing Control,” American Journal of Physical Anthropology 109 (1999): 341–363, 10.1002/(SICI)1096-8644(199907)109:3<341::AID-AJPA5>3.0.CO;2-2. 10407464

[65] P. Langer , “Lactation, Weaning Period, Food Quality, and Digestive Tract Differentiations in Eutheria,” Evolution; International Journal of Organic Evolution 57 (2003): 1196–1215, 10.1111/j.0014-3820.2003.tb00328.x. 12836835

[66] L. T. Humphrey , “Weaning Behaviour in Human Evolution,” Seminars in Cell & Developmental Biology 21 (2010): 453–461, 10.1016/j.semcdb.2009.11.003. 19914386

[67] A. Gómez‐Robles , C. Nicolaou , J. B. Smaers , and C. C. Sherwood , “The Evolution of Human Altriciality and Brain Development in Comparative Context,” Nature Ecology & Evolution 8 (2024): 133–146, 10.1038/s41559-023-02253-z. PMC10781642 38049480

[68] M. K. Kern , S. Jaradeh , R. C. Arndorfer , and R. Shaker , “Cerebral Cortical Representation of Reflexive and Volitional Swallowing in Humans,” American Journal of Physiology‐Gastrointestinal and Liver Physiology 280 (2001): G354–G360, 10.1152/ajpgi.2001.280.3.G354. 11171617

[69] S. Hamdy , D. J. Mikulis , A. Crawley , et al., “Cortical Activation During Human Volitional Swallowing: An Event‐Related fMRI Study,” American Journal of Physiology‐Gastrointestinal and Liver Physiology 277 (1999): G219–G225, 10.1152/ajpgi.1999.277.1.G219. 10409170

[70] A. Iriki , S. Nozaki , and Y. Nakamura , “Feeding Behavior in Mammals: Corticobulbar Projection Is Reorganized During Conversion From Sucking to Chewing,” Developmental Brain Research 44 (1988): 189–196, 10.1016/0165-3806(88)90217-9. 3224424

[71] V. E. Negus , “The Comparative Anatomy and Physiology of the Larynx,” The Laryngoscope 60 (1950): 516–516, 10.1288/00005537-195005000-00010.

[72] W. T. Fitch , “The Evolution of Speech: A Comparative Review,” Trends in Cognitive Sciences 4 (2000): 258–267, 10.1016/s1364-6613(00)01494-7. 10859570

[73] T. Nishimura , “Primate Vocal Anatomy and Physiology: Similarities and Differences Between Humans and Nonhuman Primates,” in The Origins of Language Revisited, ed. N. Masataka (Springer, 2020), 25–53, 10.1007/978-981-15-4250-3_2.

[74] B. de Boer and W. T. Fitch , “Computer Models of Vocal Tract Evolution: An Overview and Critique,” Adaptive Behavior 18 (2010): 36–47, 10.1177/1059712309350972.

[75] P. Lieberman , “The Evolution of Human Speech: Its Anatomical and Neural Bases,” Current Anthropology 48 (2007): 39–66, 10.1086/509092.

[76] T. Nishimura , “Developmental Changes in the Shape of the Supralaryngeal Vocal Tract in Chimpanzees,” American Journal of Physical Anthropology 126 (2005): 193–204, 10.1002/ajpa.20112. 15386289

[77] M. Knörnschild , O. Von Helversen , and F. Mayer , “Twin Siblings Sound Alike: Isolation Call Variation in the Noctule Bat, *Nyctalus noctula* ,” Animal Behaviour 74 (2007): 1055–1063, 10.1016/j.anbehav.2006.12.024.

[78] K. L. Kramer , “The Evolution of Human Parental Care and Recruitment of Juvenile Help,” Trends in Ecology & Evolution 26 (2011): 533–540, 10.1016/j.tree.2011.06.002. 21784548

